# Radiation Safety Awareness Among Non-radiology Staff at Tabuk Hospitals, Saudi Arabia

**DOI:** 10.7759/cureus.70603

**Published:** 2024-10-01

**Authors:** Siraj Fahad Wally, Sarah Ali H. Abu Sabir, Shoog M Alharbi, Ibrahim Ahmed J Albalawi, Kadi Mohsen R. Alharbi, Najd Binsulaiman, Nouf M Albalawi, Abdulrahman M Alshareef

**Affiliations:** 1 Radiology, University of Tabuk, Tabuk, SAU; 2 Medicine, University of Tabuk, Tabuk, SAU; 3 Radiology, Prince Sultan Military Medical City, Riyadh, SAU; 4 Pharmacy, University of Tabuk, Tabuk, SAU; 5 Medicine, Alfaisal University, Riyadh, SAU; 6 General Practice, Ministry of Health, Tabuk, SAU

**Keywords:** awareness, ionizing radiation, non-radiology staff, radiation safety, radiology

## Abstract

Background: Insufficient understanding of radiation safety contributes to heightened exposure vulnerability among patients and medical personnel.

Objectives: This study assessed radiation safety awareness among non-radiology staff at Tabuk hospitals, Saudi Arabia.

Methods: This cross-sectional study included 203 non-radiology staff from the King Salman Armed Forces, King Fahad Specialist, and King Khaled Hospitals in Tabuk City, Saudi Arabia. A self-administered, structured questionnaire was used. Regression analysis was used to detect variables affecting radiation safety awareness.

Results: According to Bloom's cut-off categories for knowledge, most non-radiologists at Tabuk hospitals (76%) had low awareness levels. Having a moderate-to-high knowledge level regarding radiation safety was significantly associated with being a physician (p = 0.004), having a longer length of service (p = 0.001), having attended a radiation protection and safety course (p = 0.049), and increased frequency of ordering imaging per day (p < 0.001). Gender had no significant effect on the knowledge level (p = 0.854). Multivariate regression analysis revealed that the daily frequency of ordering images was the only independent significant factor associated with having a moderate-to-high level of knowledge (OR: 6.222, 95% CI: 2.706-14.308, p < 0.001).

Conclusions: Non-radiologists in Tabuk hospitals have low awareness of radiation safety. Strong associations were noticed between awareness level and being a physician, having clinical experience, attending a radiation protection and safety course, and increasing the frequency of ordering imaging daily. Training courses about the hazards of radiation and the safety measures could lower the frequency of daily exposure to radiation.

## Introduction

The impact of ionizing radiation on human health is dangerous. Healthcare personnel and patients experience the greatest exposure to ionizing radiation during medical interventions, whether for treatment or diagnostic purposes [1]. Ionizing radiation alters the human body's chemical characteristics, leading to either cell death or impairment of cellular function, which can contribute to the onset of cancer [2].

Ensuring the safety of both the patients and the healthcare personnel is a fundamental concern in all diagnostic and therapeutic procedures that utilize ionizing radiation. Healthcare professionals who come into contact with ionizing radiation must adhere to the As Low As Reasonably Achievable (ALARA) principles, and this entails conducting scans utilizing minimal doses of ionizing radiation [3,4].

Given that many radiological exams are initiated by individuals not specialized in radiology, they must understand the radiation dosage associated with these examinations before making such requests [5]. The International Commission on Radiological Protection has implemented many preventive measures to mitigate the risk of radiation-induced cancer and other associated health issues. It is advised that the justification for all patient exposures is ensured and that efforts are made to minimize radiation doses. It is also advisable to restrict dosages [6].

The knowledge and awareness of healthcare professionals play a significant role in the danger of radiation exposure [7]. Physicians must comprehensively understand radiation dosage in routine radiation procedures and the associated dangers of exposure to provide patients with sufficient and accurate information [8].

Numerous studies have documented varying outcomes regarding the extent of awareness of radiation safety practices among non-radiology personnel [9-11]. However, there is a dearth of evidence concerning physicians' awareness of the risks posed by ionizing radiation and the possible factors related to the awareness level [12]. No prior investigations have been conducted in Tabuk hospitals to ascertain the extent of awareness explicitly. This study aimed to assess radiation safety awareness among non-radiology staff at Tabuk hospitals.

## Materials and methods

Ethical considerations

The protocol of this study obtained approval from the Research Ethics Committee of the Faculty of Medicine, University of Tabuk, Saudi Arabia (ID: UT-431-193-2024, Date: 17/3/2024). Before the data collection, all participants were informed about the study objectives and methodology, and informed consent was obtained from each participant. The participants’ data were kept confidential. 

Study design, setting, and date

This cross-sectional study was conducted at King Salman Armed Forces, King Fahad Specialist, and King Khaled Hospitals in Tabuk City, Saudi Arabia. Data collection was carried out between April and May 2024.

Eligibility criteria

The study enrolled all the non-radiology staff of both sexes in the three hospitals. Radiology staff, non-radiology staff outside Tabuk, individuals who did not agree to participate, and those with incomplete data were excluded.

Data collection tool

Each participant was given an online self-administered questionnaire, which they were instructed to complete while following the notes and directions of the data collectors. A note outlining the study's goals and requesting participant approval was included along with the questionnaire. Bloom’s cut-off categories were used for knowledge level assessment.

The questionnaire consisted of 10 questions. The first five questions collected the participants’ sociodemographic and practice-related data, while the next five questions assessed radiation safety awareness [4,12,13].

The demographic data included gender, profession, and length of service. The practice-related data included attendance on a radiation protection and safety course and times of daily ordering images. The radiation safety awareness data included knowledge about the highest radiation modalities, susceptibility to cancer after X-ray exposure, tissues having more susceptibility to injury from ionizing radiation, the safety of entrance to the computed tomography scans room, and the population groups at risk of being more affected by radiation damage.

Sample size and sampling technique

The sample size was calculated using the OpenEpi for Epidemiologic Statistics Software. The formula used for calculating the sample size of 202 is

n = (Z^2 * p * (1-p)) / E^2

Where n = desired sample size Z = Z statistic for a level of confidence (e.g., 1.96 for a 95% confidence level) p = estimated proportion of the population expressing a characteristic E = desired margin of error

We got a sample size of 202 participants based on the following criteria: 59.2% prevalence of low awareness among non-radiologists from an earlier study (14), 95% confidence interval, and 80% power of the test. The study participants were selected using a convenience sampling method. 

Statistical methods

The analysis used Statistical Product and Service Solutions (SPSS, version 26; IBM SPSS Statistics for Windows, Armonk, NY). Categorical variables (e.g., gender, profession) were summarised as frequencies and percentages. Numerical variables (e.g., the percentage of correctly answered questions) were summarized using the mean and standard deviation. The association between nominal categorical variables was done using Pearson’s chi-square test for independence of observations. In the case of association between ordinal (e.g., length of service) and nominal variables, the Cochran-Armitage test was performed. Multivariate binomial logistic regression was performed using variables with a p-value <0.1 in univariate analysis. A p-value <0.05 was adopted to indicate the significance level of the test results.

## Results

The total number of completed questionnaires was 202. More than half the participants were males (54.0%), and approximately three-fourths were physicians (76.7%). The length of service was less than one year in 27.7% of participants and one to five years in 42.6%, while much lower percentages of participants had a length of service of 6-10 years, 11-15 years, or > 16 years (19.3%, 8.4%, and 2%, respectively). Nearly half the participants attended a radiation protection and safety course (51%). About 14% of participants declared they do not order radiological imaging daily during their work. Most participants (73.3%) reported ordering imaging five times daily (Table 1).

**Table 1:** Sociodemographic and practice-related data of the respondents (total number = 202) (CT) Computed tomography

Sociodemographic and practice-related data		N (%)
Gender	Female	93 (46.0%)
	Male	109 (54.0%)
Profession	Other	47 (23.3%)
	Physician	155 (76.7%)
Duration of professional experience	<1 year	56 (27.7%)
	1-5 years	86 (42.6%)
	6-10 years	39 (19.3%)
	11-15 years	17 (8.4%)
	> 16 years	4 (2.0%)
Attended a radiation protection and safety course	No	99 (49.0%)
	Yes	103 (51.0%)
Frequency of ordering images like x-rays, CT ... etc. per day	None	29 (14.4%)
	Around 5 times	148 (73.3%)
	Around 10 times	15 (7.4%)
	More than 10	10 (5.0%)

**Figure 1:**
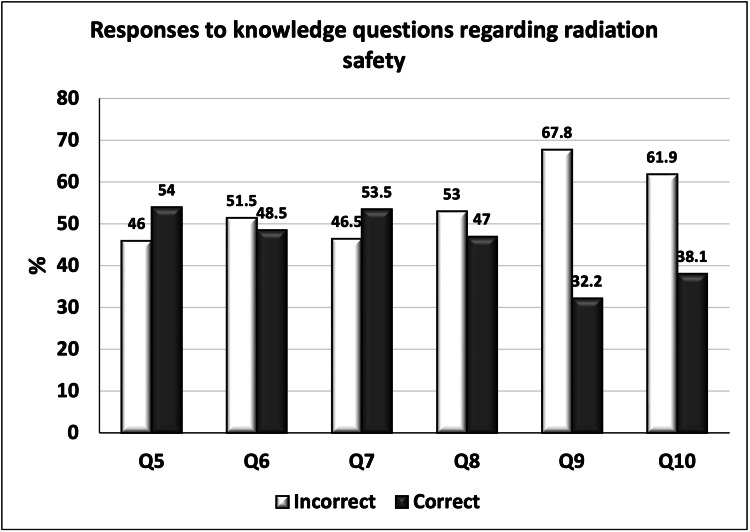
Percentages of incorrect and correct responses to the knowledge questions regarding radiation safety (total number = 202)

The participants’ responses to the questions assessing their knowledge about radiological safety are summarized in Table 2. The percentage of correct answers varied across the questions, with the highest percentage of correct answers in questions five (54%) and seven (53.5%), followed by questions six (48.5%) and eight (47%). The lowest percentages of correct answers were found in questions nine (32.2%) and 10 (38.1%; Figure 1). Answers to question 10 were correct if the participant selected children and pregnant women as the most affected group for risk of radiation exposure.

**Table 2:** Participants’ responses to questions about knowledge safety (total number = 202) (CT) computed tomography; (MRI) magnetic resonance imaging

Knowledge regarding radiation safety		N (%)
Modalities have more radiation	Computed Tomography (CT)	109 (54.0%)
	Magnetic Resonance Imaging (MRI)	48 (23.8%)
	Ultrasonography	21 (10.4%)
	X-ray	24 (11.9%)
X-ray radiation used for diagnostic imaging examinations might increase the risk of patients developing cancer in the future	No	6 (3.0%)
	Yes	98 (48.5%)
Most common complication of radiation exposure	Acute radiation syndrome	84 (41.6%)
	Infertility	10 (5.0%)
	Thyroid cancer	108 (53.5%)
Tissues are more susceptible to damage by ionizing radiation	Bone	63 (31.2%)
	Breast	95 (47.0%)
	Kidney	32 (15.8%)
	liver	12 (5.9%)
Safety of the patient's relative to enter the CT room with the patient during the imaging process	No	65 (32.2%)
	No opinion	81 (40.1%)
	Yes	56 (27.7%)
Most affected group for risk of radiation exposure	Children	80 (39.6%)
	Pregnant woman	195 (96.5%)
	Old age	142 (70.3%)

**Figure 2:**
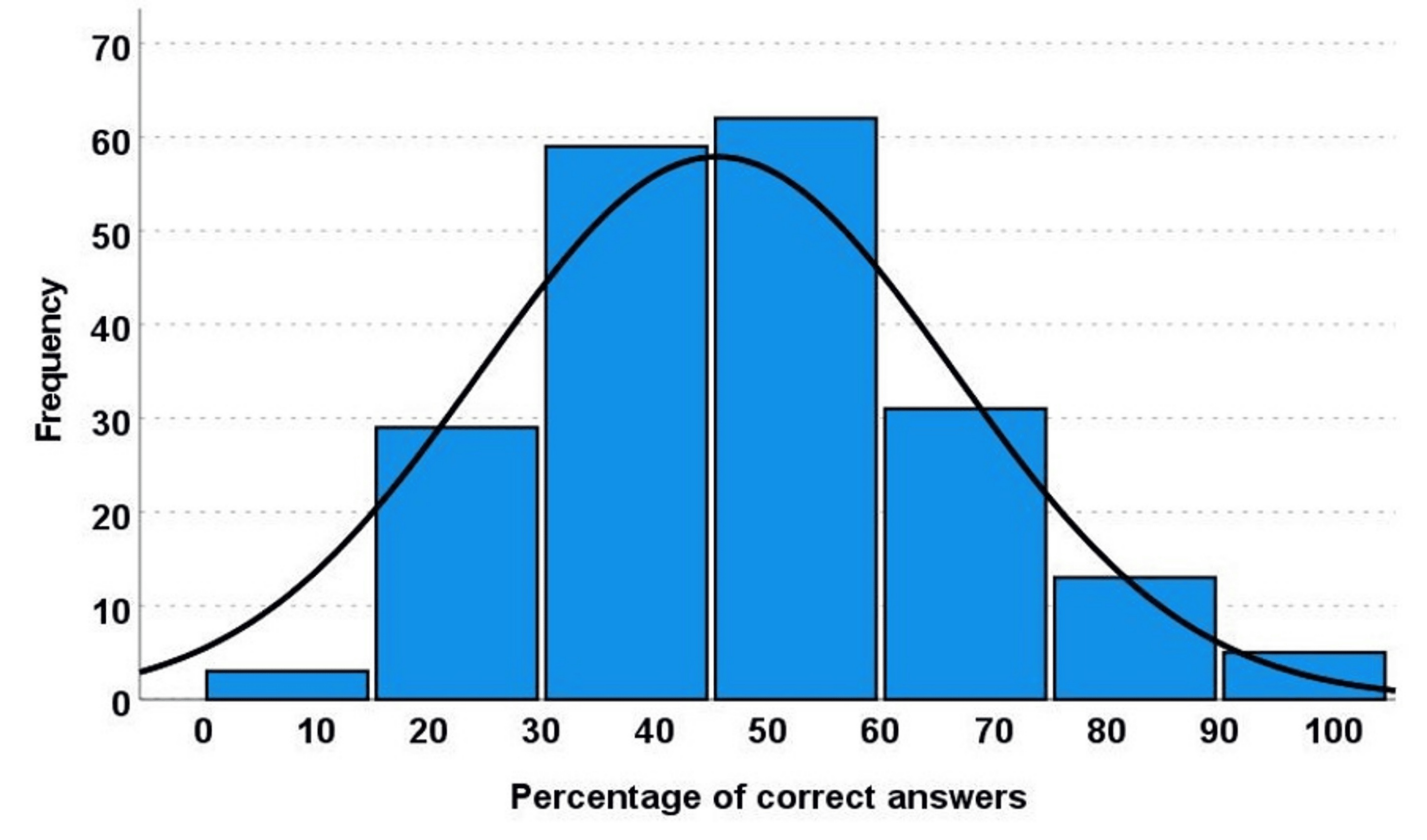
Histogram showing the percentage of the total correct responses in questions 5-10 Mean = 45.54, standard deviation = 20.88 (total number = 202)

**Figure 3:**
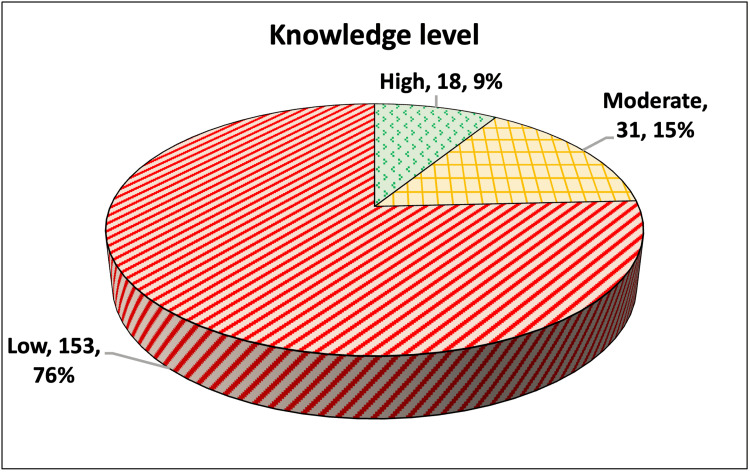
Pie chart showing the level of knowledge regarding radiation safety among the participants (total number = 202)

The percentage of correctly answered questions was calculated for each participant. The mean percentage was 45.54, with a standard deviation of 20.88 (Figure 2). Using Bloom’s cut-off categories for knowledge levels (Figure 3), we found that only 18/202 (9%) had a high level (percentage of correct answers ≥80%), 31/202 (15%) had a moderate level (percentage of correctly answered questions ≥60% and less than 80%), and 153/202 (76%) had a low level (percentage of correctly answered questions <60%).

The analysis then attempted to explore the potential effect of the participants’ characteristics on the level of knowledge. The knowledge levels “moderate” and “high” were merged into one category and compared to the low level. Having a moderate-to-high knowledge level regarding radiation safety was significantly associated with being a physician (p = 0.004), having a longer length of service (p = 0.001), having attended a radiation protection and safety course (p = 0.049), and increased frequency of ordering imaging per day (p < 0.001). Meanwhile, there was no significant association with the participants’ gender (p = 0.854) (Table 3).

**Table 3:** The association of the participants’ sociodemographic and practice-related data with the level of knowledge (total number = 202)

Sociodemographic and practice-related data		Knowledge level		p-value
		Low (n = 153)	Moderate/High (n = 49)	
Gender	Female	71 (76.3%)	22 (23.7%)	0.854
	Male	82 (75.2%)	27 (24.8%)	
Profession	Others	43 (91.5%)	4 (8.5%)	0.004*
	Physician	110 (71.0%)	45 (29.0%)	
Duration of service	<1 year	46 (82.1%)	10 (17.9%)	0.001*
	1 - 5 years	69 (80.2%)	17 (19.8%)	
	6 - 10 years	28 (71.8%)	11 (28.2%)	
	11 - 15 years	10 (58.8%)	7 (41.2%)	
	> 16 years	0 (0.0%)	4 (100.0%)	
Attended a radiation protection and safety course	No	81 (81.8%)	18 (18.2%)	0.049*
	Yes	72 (69.9%)	31 (30.1%)	
Frequency of ordering imaging per day	None	28 (96.6%)	1 (3.4%)	<0.001*
	Around 5 times	118 (79.7%)	30 (20.3%)	
	Around 10 times	7 (46.7%)	8 (53.3%)	
	More than 10	0 (0.0%)	10 (100.0%)	

To adjust for the potential confounding variables in the participants’ characteristics, a multivariate logistic regression analysis was undertaken. All the variables were entered into the model except for gender, as the p-value from the univariate analysis in Table 3 exceeded the limit of 0.1, which is usually adopted for inclusion in multivariate regression. The frequency of ordering images per day was the only independent significant factor associated with having a moderate-to-high level of knowledge (OR: 6.222, 95% CI: 2.706-14.308, p < 0.001; Table 4).

**Table 4:** Multivariate logistic regression analysis to assess the effect of participants’ characteristics on the level of knowledge B: regression coefficient; CI: confidence interval; OR: odds ratio; SE: standard error; *significant at p < 0.05.

Independent variables	B	SE	Wald test	p-value	OR	95% CI for OR
Physician (reference: others)	0.710	0.598	1.411	0.235	2.033	0.630-6.559
Attended a radiation protection and safety course	0.554	0.385	2.072	0.150	1.741	0.818-3.703
Duration of service	0.101	0.209	0.232	0.630	1.106	0.734-1.666
Frequency of ordering images per day	1.828	0.425	18.514	<0.001*	6.222	2.706-14.308

## Discussion

Physicians must understand the associated dangers of radiation exposure to provide patients with sufficient and accurate information [8,14]. Hence, this study aimed to assess the knowledge of radiation hazards among non-radiologists in Tabuk City. According to Bloom's cut-off categories, our main findings revealed a low awareness of radiation hazards among non-radiological physicians. Awareness of radiation safety was significantly associated with being a physician, having a longer service length, attending a radiation protection and safety course, and increasing the frequency of ordering imaging daily. The frequency of ordering imaging per day was the only independent significant factor associated with having a moderate-to-high level of knowledge.

Our mean knowledge percentage was 45.54 ± 20.88. Seventy-six percent of our participants had a low knowledge level. Similarly, Abdellah et al. [15] reported a mean percent score of 56.5 ± 15.2 in Egypt. Several studies revealed low awareness levels about radiation safety and hazards inside [9,11,16-19] and outside [4,8,20,21] Saudi Arabia. In Saudi Arabia, Alrefaei et al. [17] found poor awareness about radiation doses and risks in Tabuk. Similar findings were observed in Riyadh [9,11,16]. Medical interns [22], pediatricians [17], emergency physicians [20], and orthopedics operating room personnel [19] had suboptimum awareness about radiation hazards. On the contrary, a high degree of awareness about radiation hazards and safety was demonstrated by an earlier study in Italy [23]. Furthermore, in Iran, a commendable level of radiation safety awareness was observed among the personnel in 18 hospitals [24]. The differences between these findings depend on the diversity of the factors affecting poor knowledge, such as attendance of safety training courses, and inadequacy of safety equipment. Salama et al. [18] spotted that several healthcare facilities in Saudi Arabia were deficient in crucial radiation protection equipment including lead glasses and shields. Therefore, it was imperative to perform additional research to evaluate the extent of protective measures implemented in each institution, ensuring that the requisite safeguards were duly observed.

There was no significant association with the participants’ gender. However, Najjar et al. [9] noticed that male physicians exhibited higher awareness levels than their female counterparts. The authors claimed that the discrepancy in knowledge can be attributable to the differential interest levels between men and women in the technical aspects of certain subjects [9].

The International Commission on Radiological Protection released recommendations designating the ovaries and testes, bone marrow, and eye lens as the organs most susceptible to radiation [25]. The findings of this study revealed a significant proportion of the participants, nearly half of them, exhibited an inability to identify the organ with the highest level of sensitivity. Saeed et al. [26] observed a lack of knowledge regarding the gonads being the organ most vulnerable to the effects of ionizing radiation. A systematic review showed that physicians and patients exhibited insufficient understanding of the potential cancer risk and radiation dosage related to computed tomography scans [27].

The moderate-to-high awareness of radiation safety was significantly associated with longer service length. Gecaga et al. [28] and Puri et al. [20] observed that doctors with extensive experience better understand the risks associated with radiation commonly used in multidetector computed tomography scans. Consequently, these experienced doctors are more inclined to consider the radiation dose received by patients and conduct a thorough risk-benefit analysis before proceeding with the scans [20].

We reported a significant association between knowledge level and being a physician. This is in contrast to Alghamdi et al. [29] who revealed no statistically significant disparity regarding this point among different healthcare professionals.

Several studies [4,26,30] showed divergent findings suggesting that the practitioner's experience level does not necessarily correspond to an enhanced understanding of radiation exposure. Szarmach et al. [4] observed a deficiency in the knowledge level of workers who have been serving for 6-10 years and over 16 years. The observed phenomenon may be attributed to the limited accessibility of radiological protection training programs and the resistance exhibited by experienced personnel toward modifying established professional practices. However, Saeed et al. [26] noticed a negligible association between physicians' awareness of radiation hazards and their level of experience. The selection of physicians with varying specialties for participation in their study was conducted randomly. This finding suggests that there may be a disparity in the level of interest in medical radiological exposure between physicians who participated in the survey and those who did not. Consequently, this discrepancy could impact the validity and reliability of the results obtained. Nevertheless, our results revealed that senior physicians were more likely to provide accurate responses to radiation dosage inquiries when referral standards were consistently utilized [28,30].

A statistically significant association was observed between the participants' knowledge level and their attendance at training events and refresher courses on radiation protection. Several earlier studies [14,20,22] found a significant relationship between the low awareness of radiation safety and the limited participation of physicians in training sessions or refresher courses on radiation protection. Foley et al. [31] reported insufficient training in radiation safety among medical personnel, leading to a decline in their understanding of what needed improvement. Furthermore, it has been observed that cardiologists' knowledge could be more optimal, where targeted training efforts can significantly enhance it [32]. Additionally, the rules set forth by the European Community advocate for including radiation protection courses in the foundational training programs for dentists, surgeons, and all physicians’ courses [33].

According to Krille et al. [8], there was an increase in the percentage of accurate answers on questionnaires when students attended radiation protection classes during residency or medical school. The implementation of suitable educational measures had the potential to effectively mitigate the deficiency in understanding and application of radiation safety principles. However, this study suggested a need for certain corrective steps to be adopted among pediatricians who continue to downplay the significance of radiation doses and associated hazards. These measures included enhancing the curriculum of radiation prevention courses during medical school and promoting the relevance of radiation protection within the medical community. Ensuring that physicians possess adequate knowledge is a crucial aspect of radiation protection, as it enables them to perform a comprehensive and precise evaluation of the risk-benefit ratio when contemplating the utilization of radiation-based diagnostic procedures. The primary responsibility of pediatric radiologists is to disseminate information and educate their professional peers regarding radiation protection. This includes raising awareness among colleagues about the potential hazards associated with radiation exposure and imparting knowledge on effective measures to mitigate these risks [8].

Continuous medical education programs are essential for individuals occupationally exposed to ionizing radiation to raise awareness about radiation protection practices. Awareness is widely acknowledged as a crucial initial stage before individuals can adopt, comply with, and adhere to a national regulatory framework [34].

The frequency of ordering images per day was a significant factor associated with sufficient knowledge. According to Najjar et al. [9], the excessive utilization of medical imaging can be ascribed to the inclination of certain patients to demand such procedures while rejecting clinical diagnosis. However, physicians who possess limited awareness regarding other approaches that can yield comparable outcomes to ionizing imaging could excessively utilize medical imaging. Additionally, it is worth noting that certain medical practitioners may occasionally order an X-ray examination without doing a comprehensive and meticulous physical assessment. In certain instances, clinicians may have challenges arriving at a conclusive diagnosis, prompting the referring physician to seek imaging procedures to narrow potential diagnoses.

Thus, it is imperative to provide targeted education to the public and patients to mitigate this conflict. Moreover, physicians who prescribe radiological imaging must possess sufficient clinical expertise and specialized knowledge regarding these procedures and their potential effects on specific populations.

Limitations

This study employed a cross-sectional design and did not establish a causal association between being a non-radiologist and radiation safety awareness. The potential for recollection bias was present in the self-administered questionnaires. The study was conducted in a specific region, which may not be representative of the broader community of non-radiologists in Saudi Arabia. Nevertheless, the outcomes of our research facilitate a more extensive investigation including various regions inside Saudi Arabia.

## Conclusions

A significant proportion of the participants, primarily physicians with varying lengths of service, have a low level of knowledge regarding radiological safety. Only a small percentage demonstrated a high level of knowledge, while the majority fell into the low knowledge category. The participants who attended a radiation protection and safety course fared slightly better in terms of knowledge levels. It also highlights the areas where participants struggled the most in terms of correct answers, particularly regarding the risk of radiation exposure to children and pregnant women.

Further research or interventions may be needed to enhance the understanding and awareness of radiological safety among healthcare professionals, especially in areas where knowledge levels were found to be lacking. Targeted educational programs or refresher courses could potentially help improve overall knowledge levels and ultimately contribute to better patient care and safety in radiology practices.

## Appendices

Data collection tool

Demographics

· Gender

- Male

- Female

1. What is your profession?

- Physician 

- Nurse 

- X-ray diagnostics technician

- Other 

2. What is the length of your service?

- Less than 1 year

- 1-5 years

- 6-10 years

- 11-15 years

- More than 16 years

3. How frequent is your contact with imaging examinations of patients?

- None

- Several times a month

- Several times a week

- Several times a day

4. Do you think that X-ray radiation doses used for diagnostic imaging examinations might increase the risk of patients developing cancer in the future?

- No opinion

- Yes 

- No

5. Identify the patient’s radiation protection measures you are aware of:

- None

- Lead aprons

- Shields

- Distance from the source of radiation

- Time of exposure

- Collimation of the radiation beam

6. What is the annual whole-body dose limit for a patient? (As a guide: the limit for classified radiation workers is 20 mSv: 1990 regulations)

- 150 mSv

- 100 mSv

- 50 mSv

- 20 mSv

- 5 mSv

- 0.5 mSv

- No limit

- Don't know

7. Please score the following 4 organs in order of radiation sensitivity:

1 = Least radiosensitive

4 = Most radiosensitive

Bladder:

Gonads:

Kidney:

Stomach:

The questionnaire was developed based on similar published studies by Szarmach et al. [4] and Quinn et al. [12].
